# The Impact of 16α-[^18^F]-fluoro-17β-Estradiol Positron Emission Tomography on the Staging and Management of Invasive Lobular Carcinoma of the Breast: A Case Series

**DOI:** 10.1016/j.adro.2026.102209

**Published:** 2026-09-10

**Authors:** Alexander E. Crum, Alexandria Oliver, Marshall Harrell, Benjamin Facer, Therese Y. Andraos, Rebekah Young, Jacob Eckstein, Emmanuel Appiah-Kubi, Bridget Oppong, Arya Roy, Margaret Gatti-Mays, Sachin R. Jhawar, Sasha Beyer

**Affiliations:** a Department of Radiation Oncology, The Ohio State University Wexner Medical Center, Columbus, Ohio; b Department of Radiology, The Ohio State University Wexner Medical Center, Columbus, Ohio; c Department of Surgery, The Ohio State University Wexner Medical Center, Columbus, Ohio; d Department of Medical Oncology, The Ohio State University Wexner Medical Center, Columbus, Ohio

## Abstract

**Purpose:**

Invasive lobular cancer (ILC) poses diagnostic challenges, often leading to upstaging at surgery and the potential for suboptimal initial management. 16α-[^18^F]-fluoro-17β-estradiol positron emission tomography (FES-PET) may enhance the initial staging and management of newly diagnosed locally advanced ILC. This case series explores FES-PET’s potential role in optimizing the management of newly diagnosed locally advanced ILC.

**Methods and Materials:**

An institutional retrospective case series of 4 patients with ILC, newly diagnosed between April 2024 and April 2025, was conducted. Three patients underwent FES-PET, which altered or supported management, and 1 patient did not undergo FES-PET but was upstaged at the time of surgery. Data on patient demographics, standard-of-care, FES-PET imaging, and pre- and post-FES-PET management plans were collected.

**Results:**

In case 1, who had 100% estrogen receptor (ER)–positivity, FES-PET detected supraclavicular nodal (maximum standardized uptake value [SUV_max_] 3.3) and chest wall avidity (SUV_max_ 5.4) after neoadjuvant chemotherapy and multiple resections, prompting extended radiation volumes and a sequential boost to the supraclavicular nodes and chest wall. Case 2 had 95% ER-positive disease, and FES-PET detected osseous metastases (SUV_max_ 3.5), shifting to a metastatic management plan with systemic therapy. Case 3 had 100% ER-positive disease; FES-PET showed no metastatic disease after adjuvant chemotherapy for pT3N3a disease, prior to radiation. Case 4 was a patient with 100% ER-positive ILC, and with contemporary imaging alone was upstaged during surgery from cT2N1M0 to pT3N3a.

**Conclusion:**

FES-PET results altered radiation dose and volumes, shifted treatment to systemic therapy, and supported the initial local treatment plan in cases 1 to 3. In case 4, baseline FES-PET may have identified more extensive disease before surgery, potentially reducing unexpected postoperative upstaging or subsequent treatment modification. FES-PET may help refine staging and treatment planning in selected patients with ER-positive locally advanced ILC; however, our study is limited by its retrospective design and limited number of patients. Prospective studies are warranted to determine whether upfront FES-PET changes management or improves clinical outcomes.

## Introduction

Invasive lobular carcinoma (ILC) represents 10% to 15% of all new breast cancer (BC) diagnoses, of which 80% are estrogen receptor (ER)-positive.^1^ Despite advances in imaging, ILC remains a diagnostic challenge because of its weak enhancement, linear growth pattern, and lack of lymph node (LN) architecture disruption in the setting of metastasis.^2^ Contemporary imaging for initial workup and restaging of ILC often underestimates the burden of disease.^3^^,^^4^ These patients are often upstaged at the time of surgery and may receive suboptimal initial management. 16α-[^18^F]-fluoro-17β-estradiol (FES) positron emission tomography (PET) has shown utility in the assessment of ER-positive disease.^5^^,^^6^ Given the diagnostic challenge of ILC, FES-PET may have a potential role during initial workup and restaging. This study was approved by the institutional review board (OSU 25046), and all patient data have been deidentified in accordance with Health Insurance Portability and Accountability Act regulations. Here, we present a case series elucidating the potential role of FES-PET in the staging and multidisciplinary management of ILC.

## Cases

### Case 1

A 77-year-old female with clinical stage IIIB, cT4bN0, pathologic stage IIB, ypT3N0, ER-positive 100%, progesterone receptor (PR)-negative, and human epidermal growth factor receptor 2 (HER2)-negative ILC underwent FES-PET prior to radiation, after receiving paclitaxel, doxorubicin, and cyclophosphamide (ACT) neoadjuvant chemotherapy, with a partial response of the primary tumor and resolution of skin involvement, followed by lumpectomy with a positive margin, re-resection with a positive margin, and subsequent mastectomy with a positive margin. Preoperative mammography; breast and axillary ultrasounds; computed tomography (CT) of the chest, abdomen, and pelvis (CAP); bone scintigraphy (BS); magnetic resonance imaging (MRI); and [^18^F]fluorodeoxyglucose (FDG)-PET showed focal breast disease with enhancement extending to the skin and pectoralis and no evidence of nodal or distant involvement. Postoperative imaging demonstrated enhancement at the postmastectomy site with difficulty in discerning residual disease from postoperative enhancement and no evidence of nodal involvement (Fig. 1A, B). FES-PET revealed supraclavicular (SCL) avidity (maximum standardized uptake value [SUV_max_] 3.3) and left chest wall (CW) avidity (SUV_max_ 5.4) (Fig. 2A-D), concerning for residual disease and occult nodal involvement, upstaging the patient to clinical stage IIIC, ycT3N3c. A 1000 cGy in 5 fraction sequential boost to residual CW disease and FES-PET–avid SCL LN (Fig. 3A-D) was subsequently included in her radiation treatment plan. She has had no evidence of progression or metastatic disease at 1-year posttreatment follow-up and continues on anastrozole (AI) and zoledronic acid (BSP), after not tolerating 4 months of abemaciclib (CDK4/6i).

**Figure 1:**
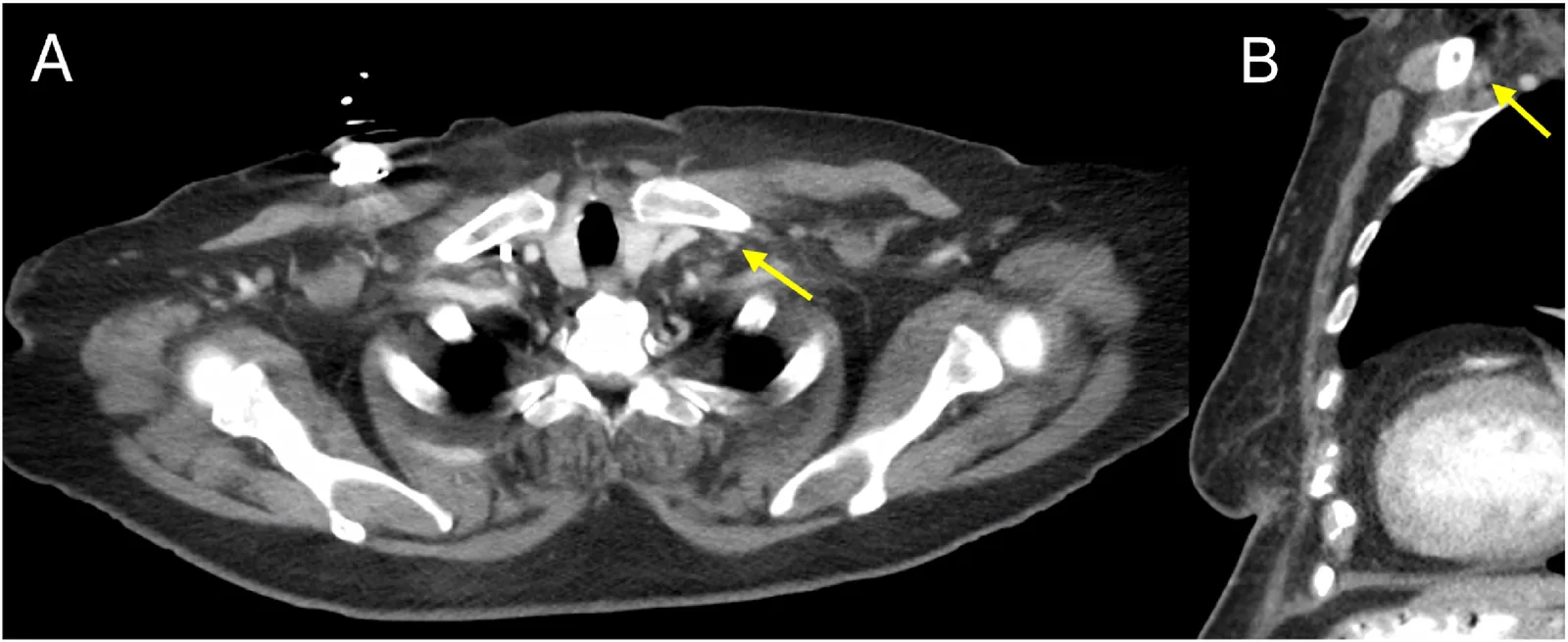
Case 1. (A) Computed tomography (CT) scan of the chest with contrast demonstrating nonenlarged supraclavicular (SCL) lymph nodes (LNs) in axial (A1) and sagittal (A2) sections. The yellow arrow in A1 designates the nonenlarged SCL LNs in an axial section of a CT chest with contrast. The yellow arrow in A2 designates the nonenlarged SCL LNs in a sagittal section of a CT chest with contrast. Given the lymph node was nonenlarged and had nonconcerning morphology, the lymph node was initially thought to be noninvolved.

**Figure 2:**
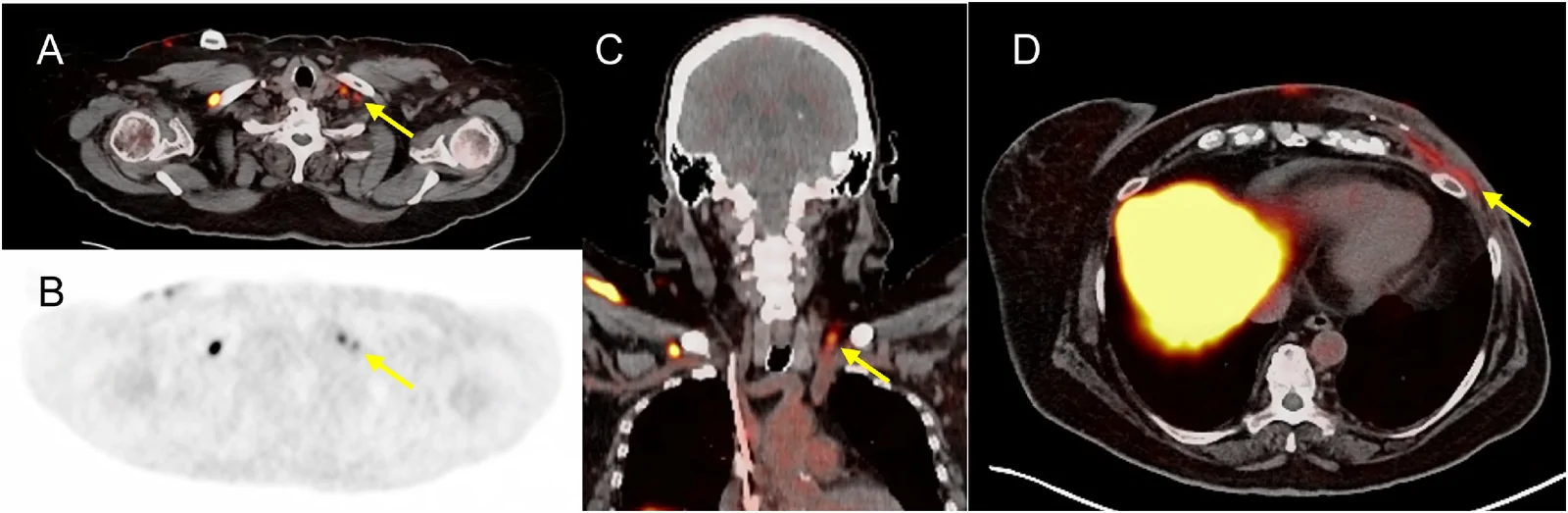
Case 1. FES-PET avidity (SUV_max_ 3.3) at the left SCL nodal region in axial (A, B) and coronal (C) sections, and FES-PET avidity at the left chest wall (CW) surgical site in axial section (D). The yellow arrows in B1-B3 designate FES-PET avidity (SUV_max_ 3.3) at the left SCL nodal region in axial (A, B) and coronal (C) sections, and the yellow arrow in D designates FES-PET avidity at the left CW surgical site in axial section. Given the increase in uptake, there was concern for occult SCL lymph node involvement and residual CW disease. The right-sided vessel and CW avidity are because of tracer administration. *Abbreviations:* FES-PET = 16α-[^18^F]-fluoro-17β-estradiol positron emission tomography; SCL = supraclavicular; SUV_max_ = maximum standardized uptake value.

**Figure 3:**
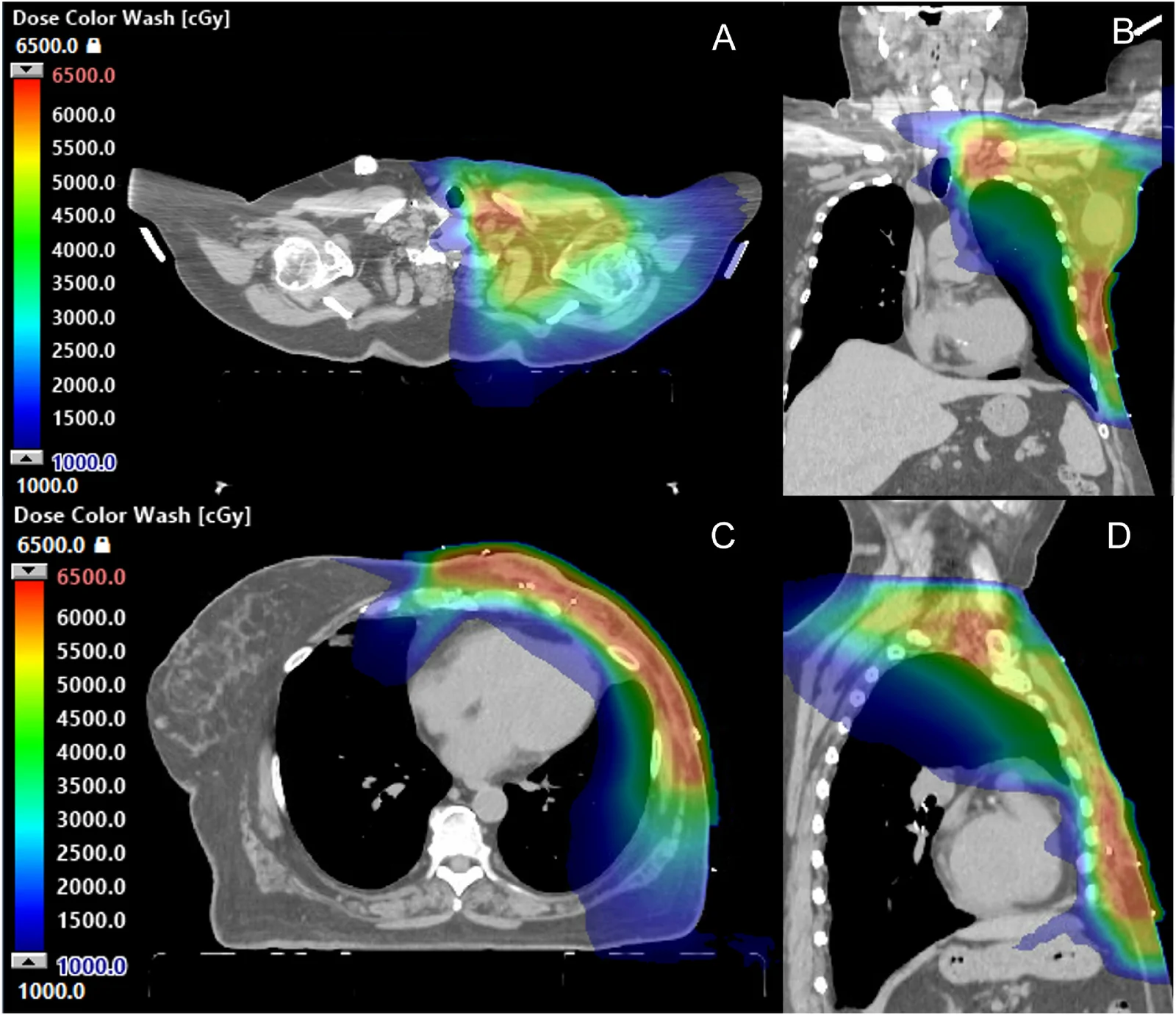
Case 1. Left CW and regional nodal irradiation 5000 cGy in 25 fractions with 1000 cGy in 5 fractions sequential boost to CW avidity and FES-PET–avid SCL LN treatment plan in axial (A, C), coronal (B), and sagittal (D) views. The dose color wash treatment plan, windowed from 1000 cGy to 6500 cGy, demonstrates prescription dose volume coverage of 5000 cGy to the CW and comprehensive regional LNs, indicated by the green/yellow to red color spectrum color wash. The sequential boost prescription dose volume, 1000 cGy sequential boost to 6000 cGy total dose, to the FES-PET–avid CW and SCL regions is indicated by the red dose color wash in C1-C4. *Abbreviations:* CW = chest wall; FES-PET = 16α-[^18^F]-fluoro-17β-estradiol positron emission tomography; LN = lymph node; SCL = supraclavicular.

### Case 2

A 72-year-old female completed FES-PET for initial workup and staging of an ER-positive (91%-100%), PR-positive, HER2-negative invasive mammary carcinoma with ductal and lobular features on outside pathology review and ILC on internal pathology review. Before FES-PET, she had completed mammography, breast ultrasound, CT CAP, BS, and MRI, which detected a breast mass, bulky level 1 to 3 axillary lymphadenopathy, asymmetric internal mammary LNs, and focal osseous enhancement of 2 right anterior ribs, interpreted as suggestive of posttraumatic pathology (Fig. 4A, B), consistent with clinical stage IIIC, cT3N3bM0. FES-PET was performed given the increased BS uptake within the right anterior fifth rib, which revealed avid subpectoral, axillary (SUV_max_ 16.6), and internal mammary LNs (SUV_max_ 8.5), avid fifth rib metastasis (SUV_max_ 3.5), and diffuse osseous metastases (SUV_max_ 8.8) (Fig. 4C-E). Subsequent biopsy of a femoral osseous lesion demonstrated metastatic BC. The patient’s treatment plan was changed from curative intent multimodality therapy to metastatic first-line therapy with AI-BSP-CDK4/6i.

**Figure 4:**
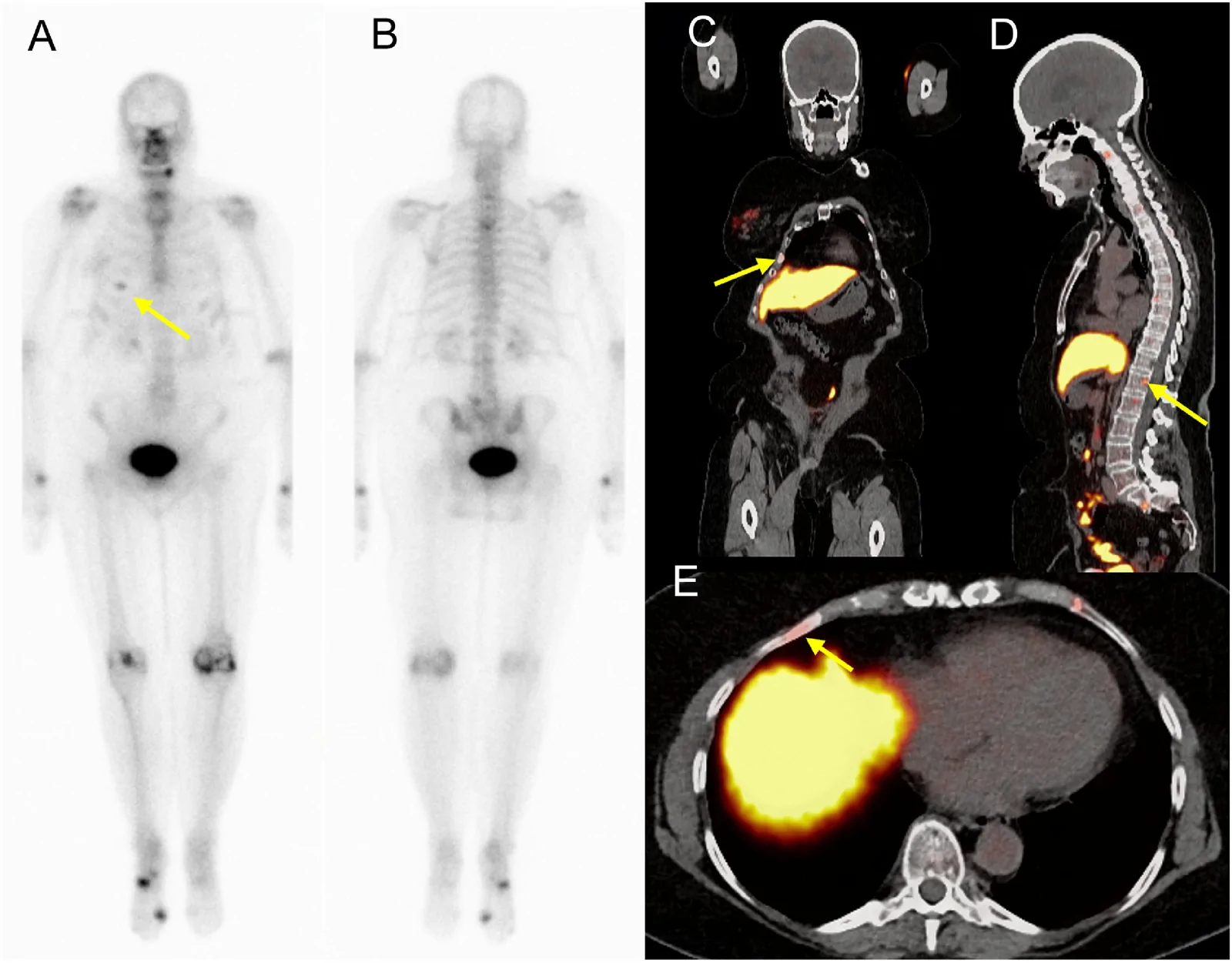
Case 2. Bone scintigraphy in anterior (A) and posterior (B) views demonstrating uptake in the right anterior fifth rib, interpreted as suggestive of a posttraumatic process. (C-E) FES-PET demonstrating right anterior fifth rib avidity (SUV_max_ 3.5) in coronal (C) and axial (E) views and avidity throughout multiple levels of the osseous spine (D). The yellow arrow in D1 designates increased uptake in the right fifth rib, interpreted as suggestive of a posttraumatic process. The yellow arrows in C and E designate increased FES-PET uptake at the right fifth rib, concerning for metastatic disease. The yellow arrow in D designates increased FES-PET uptake in a lumbar vertebral body, suggestive of metastatic disease. *Abbreviations:* FES-PET = 16α-[^18^F]-fluoro-17β-estradiol positron emission tomography; SUV_max_ = maximum standardized uptake value.

### Case 3

A 59-year-old female with pathologic stage IIIC, pT3N3a, ER-positive 100%, PR-positive, HER2-negative ILC managed with mastectomy, axillary LN dissection (ALND), and adjuvant ACT underwent FES-PET for postchemotherapy restaging. Previous CT CAP and BS prior to chemotherapy reported no evidence of disease. FES-PET showed no evidence of disease. After FES-PET, she proceeded with adjuvant CW radiation and regional nodal irradiation 5000 cGy in 25 fractions with a sequential scar boost of 1000 cGy in 5 fractions. She continues on BSP and has no evidence of recurrence 5 months after FES-PET.

### Case 4

A 64-year-old female with ER-positive 100%, PR-positive, HER2-negative ILC completed mammography, breast ultrasound, MRI, and CT CAP demonstrating multicentric breast masses, slightly enlarged, asymmetric level 1 axillary LNs, and no level 2 or 3 axillary lymphadenopathy. She was determined to be clinical stage IIA, cT2N1M0. She underwent mastectomy and sentinel LN biopsy, which demonstrated a 7.8-cm tumor and 4 of 4 positive LNs. She then received adjuvant ACT. A delayed ALND was performed with 11 of 12 LNs involved. Her surgical pathology was consistent with pathologic stage IIIC, pT3N3a. She was treated with CW radiation and regional nodal irradiation, 4256 cGy in 16 fractions, with a sequential boost to the mastectomy scar of 1000 cGy in 4 fractions. She continues on AI-BSP-CDK4/6i.

## Discussion

ILC remains a diagnostic challenge during initial staging and restaging because current standard-of-care (SOC) baseline imaging often underestimates the extent of disease.^3^, ^4^, ^5^ As a result, patients may receive initial suboptimal management. Here, we described several cases in which FES-PET changed or supported clinical decision making as well as a case in which baseline FES-PET may have been beneficial, underscoring the importance of accurate staging prior to treatment initiation.

Cases 1 to 3 demonstrate FES-PET changing or further supporting the decision for management. In case 1, after FES-PET showed ER avidity in nonenlarged, non-FDG–avid SCL LNs and residual CW disease, the patient’s radiation plan was modified to include a sequential 1000 cGy in 5 fraction boost to the CW disease and SCL nodes. Case 2 describes the utility of FES-PET during initial workup, which identified osseous metastatic disease that was not identified on previous SOC imaging. The patient’s management was transitioned to palliative systemic therapy alone. In case 3, the FES-PET that was ordered after modified radical mastectomy, ALND, and adjuvant chemotherapy (given extensive locoregional involvement and concern for residual or occult disease) showed that there was no evidence of disease, which supported the initial plan for local therapy. In case 4, baseline FES-PET may have identified more extensive disease prior to surgery, potentially reducing unexpected postoperative upstaging or subsequent treatment modification.

Retrospective analyses have reported pathologic ILC upstaging despite SOC imaging.^3^^,^^4^ At the time of surgery, women with ILC were observed to have a greater number of positive LNs (2.79 vs 2.26; *P* = .009) and were more frequently upstaged to at least pathologic stage IIIA compared with patients without lobular histology (30.9% vs 17.4%; *P* = .007).^3^ Another analysis including patients with newly diagnosed stage I to III ILC and preoperative axillary imaging including mammography, MRI, and/or ultrasound reported 70.5% of patients being clinically node negative; however, 62% were pathologically node negative (*P* = .03), and 38% of pN2/N3 patients were initially categorized as cN0.^4^

FES-PET has shown superiority to contemporary imaging in the evaluation of ER-positive recurrent or metastatic disease.^5^, ^6^, ^7^ FES-PET has been shown to have a significant advantage in the detection of ER-positive ILC bone metastases compared with FDG-PET (*P* = .05), and in the posttreatment setting, there is selectivity for ER-positive disease and decreased posttreatment background inflammation uptake compared with FDG-PET.^5^^,^^7^ A prospective multicenter trial demonstrated an FES-PET/CT sensitivity of 95% (95% CI, 89%-97%), specificity of 80% (95% CI, 66%-89%), positive predictive value of 93% (95% CI, 87%-96%), and negative predictive value of 85% (95% CI, 72%-92%) for ER-positive metastatic disease.^8^ FES-PET is now recommended during the workup for recurrent or metastatic ILC in the National Comprehensive Cancer Network guidelines.^9^

Ryu et al^10^ reported that FES-PET imaging can lead to management changes in up to 35% of patients with recurrent or metastatic BC. We hypothesize that this impact may be even more pronounced in newly diagnosed ILC cases. Baseline, treatment-naïve FES-PET may be of particular benefit in radiation treatment planning because additional information regarding suspicious LNs or identification of occult disease prior to neoadjuvant chemotherapy and surgery may change management to include extended radiation volumes or dose escalation. However, this case series is limited by its retrospective design, small sample size, selection bias in FES-PET use, lack of systematic comparison with conventional imaging, and short clinical follow-up.

## Conclusion

FES-PET is an emerging technology that may help refine staging and treatment planning in selected patients with ER-positive locally advanced ILC. Here, we presented a case series demonstrating the use of FES-PET in patients with ILC and the resultant decision-making, multidisciplinary management, and treatment planning, as well as a case demonstrating significant pathologic upstaging of ILC at the time of surgery despite SOC imaging alone. Prospective studies are warranted to determine whether upfront FES-PET changes management or improves clinical outcomes.

## Disclosures

Alexander Crum reports financial support was provided by American College of Radiation Oncology. If there are other authors, they declare that they have no known competing financial interests or personal relationships that could have appeared to influence the work reported in this paper.
